# Supplementary material for: Estimating turnover and industry longevity of Canadian sex workers
Source: PLoS One. 2024 Mar 27;19(3):e0298523. doi: 10.1371/journal.pone.0298523 (PMC10971545; doi:10.1371/journal.pone.0298523)
Supplement: S1 Appendix — (DOCX) [file pone.0298523.s003.docx]

A comparison of data collection techniques

This article describes results based on ads collected from one classified advertising site, Site 3, described in previous work [1,2]. Data collection involved downloading ads based on discovered ad URLs. For the site used for this study there were two different ways in which these URLs could be discovered.

One method involved identifying portal pages on the site and polling these pages to identify ad URLs [see downloaders/2022 in 3]. Portal pages were segmented by region and type of service (e.g. “female-escorts”). For an ad to appear in a portal page an advertiser has to “bump” the ad so that it is visible. One difficulty with this method is that there are potentially a very large number of portal pages to download. For this study, 184 pages were checked for ad URLs covering all ad categories and regions. Another difficulty is that, when many advertisers are posting ads, the length of time that one ad is visible on the portal page can be minimal. In an area and service category with a lot of activity an ad may be visible on the main page for only 30 minutes. The solution to this problem was to download frequently. For the purposes of this study all portal pages were checked every 10 minutes. While this captured a very large number of ads it may have missed some ads when the sites were especially busy.

Another method is to take advantage of data that is provided by the site operator to discover ad URLs. Search engines use certain standard files such as *robots.txt* and *sitemap.xml* for this purpose. The *sitemap.xml* file uses a standard XML format [4] and is read by search engine bots when crawling a site. The *sitemap.xml* file can have direct links to website pages or can provide links to other sitemap files. In the case of Site 3 the structure for ad links was *sitemap.xml > sitemap_itemN.xml* where N can be any natural number. On Site 3 the number of files containing ad URLs was typically 180 to 190 in any given day. These files were updated daily at least once per day. When identifying ads using these sources the sitemaps were downloaded between one and three times per day and searched for new ad URLs.

It was not clear whether the ads collected using sitemaps were the same as those collected by polling portal pages on the site. To resolve this question, in addition to portal pages, sitemaps were downloaded and the links contained in the sitemaps were compared with links found in the portal pages to see whether they were the same. Given that sitemaps are generated by the website operator I wanted to understand how long ad URLs were visible in the sitemaps to see if this might have an effect on calculations regarding when an advertiser was active. It was possible that some ad links could be archived when the advertiser was not active.

To clarify these questions, the first and last date that links from each source were visible was collected and stored in a MariaDB database [5] and queries were made to identify how many URLs were different and how long, in general URLs were visible. Table 1 shows the number of ad URLs found in portal pages showing how many were also visible in sitemaps. It shows that while there is an 82.0% (N=769268) overlap between the two sources, 17.9% (N=168168) of the ad links found in the portal pages were not found in the sitemaps. Table 2 shows how many of the ad URLs found in sitemaps matched those found portal pages. The sitemaps also contained a large number of URLs (26.0%, N=276237) that were never found in portal pages.

**Table 1:** Comparison of ad URLs found in portal pages with ad URLs found in sitemaps.

| in sitemap? | N | percent |
| --- | --- | --- |
| found | 769268 | 82.0% |
| not found | 168168 | 17.9% |

**Table 2:** Comparison of ad URLs found in sitemaps with ad URLs found in portal pages.

| in a portal page? | N | percent |
| --- | --- | --- |
| found | 769268 | 73.6% |
| not found | 276237 | 26.4% |

The length of time urls were visible differed considerably between the two sources. Figure 1 shows the distributions of days visible for matched URLs from both sources (N=785457). URLs found in sitemaps tended to be visible longer compared to their matched counterparts found in portal pages (mean difference sitemap vs portal page 32.4 days, SD 70.2).

**Figure 1:** Days visible for URLs discovered in portal pages and sitemaps.
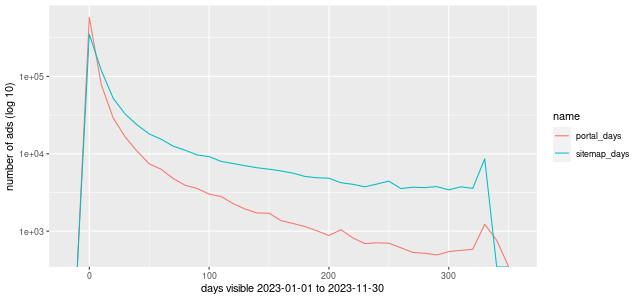


While there is considerable overlap between the two sources, and in previous work that used sitemaps as the starting point for data collection [2] all discovered URLs could be downloaded without error, it is troubling that there are large numbers of ads missing from both sources. Furthermore, can we say that the generally longer lifespan of URLs in the sitemaps reflects active advertisers? For the purposes of this study I felt that polling the portal pages was a more naturalistic approach which, for population studies, was more appropriate as it reflected when advertisers were active.

# References

1. Kennedy L. The silent majority: The typical Canadian sex worker may not be who we think. PloS one. 2022;17: e0277550–e0277550.

2. Kennedy L. Estimating turnover and industry longevity of Canadian sex workers. SocArXiv; 2023. doi:10.31235/osf.io/qr75c

3. Population Project. Pop Downloader. 2021. Available: https://gitlab.com/population.project.2021/pop-downloader-public

4. sitemaps.org. sitemaps.org - Protocol. [cited 22 Dec 2023]. Available: https://www.sitemaps.org/protocol.html

5. MariaDB, Widenius M. MariaDB. 2017. Available: https://mariadb.com/kb/en/about-mariadb-software/
