# Supplementary material for: Estimating turnover and industry longevity of Canadian sex workers
Source: PLoS One. 2024 Mar 27;19(3):e0298523. doi: 10.1371/journal.pone.0298523 (PMC10971545; doi:10.1371/journal.pone.0298523)
Supplement: Attachment — Submitted filename: Response to Reviewers.docx [file pone.0298523.s004.docx]

Response to Reviewers PONE-D-23-35872 Estimating turnover and industry longevity of Canadian sex worker

PLOS ONE

Dear Dr. Kennedy,

Thank you for submitting your manuscript to PLOS ONE. After careful consideration, we feel that it has merit but does not fully meet PLOS ONE’s publication criteria as it currently stands. Therefore, we invite you to submit a revised version of the manuscript that addresses the points raised during the review process.

Please submit your revised manuscript by Feb 11 2024 11:59PM. If you will need more time than this to complete your revisions, please reply to this message or contact the journal office at plosone@plos.org. When you're ready to submit your revision, log on to <https://www.editorialmanager.com/pone/> and select the 'Submissions Needing Revision' folder to locate your manuscript file.

Please include the following items when submitting your revised manuscript:

- A rebuttal letter that responds to each point raised by the academic editor and reviewer(s). You should upload this letter as a separate file labeled 'Response to Reviewers'.
- A marked-up copy of your manuscript that highlights changes made to the original version. You should upload this as a separate file labeled 'Revised Manuscript with Track Changes'.
- An unmarked version of your revised paper without tracked changes. You should upload this as a separate file labeled 'Manuscript'.

If you would like to make changes to your financial disclosure, please include your updated statement in your cover letter. Guidelines for resubmitting your figure files are available below the reviewer comments at the end of this letter.

If applicable, we recommend that you deposit your laboratory protocols in [protocols.io](http://protocols.io/) to enhance the reproducibility of your results. Protocols.io assigns your protocol its own identifier (DOI) so that it can be cited independently in the future. For instructions see: [https://journals.plos.org/plosone/s/submission-guidelines#loc-laboratory-protocols](https://journals.plos.org/plosone/s/submission-guidelines#loc-laboratory-protocols). Additionally, PLOS ONE offers an option for publishing peer-reviewed Lab Protocol articles, which describe protocols hosted on [protocols.io](http://protocols.io/). Read more information on sharing protocols at <https://plos.org/protocols?utm_medium=editorial-email&utm_source=authorletters&utm_campaign=protocols>.

We look forward to receiving your revised manuscript.

Kind regards,

Hamid Sharifi
Academic Editor
PLOS ONE

Journal Requirements:

When submitting your revision, we need you to address these additional requirements.

1. Please ensure that your manuscript meets PLOS ONE's style requirements, including those for file naming. The PLOS ONE style templates can be found at
<https://journals.plos.org/plosone/s/file?id=wjVg/PLOSOne_formatting_sample_main_body.pdf> and
<https://journals.plos.org/plosone/s/file?id=ba62/PLOSOne_formatting_sample_title_authors_affiliations.pdf>

2. Please review your reference list to ensure that it is complete and correct. If you have cited papers that have been retracted, please include the rationale for doing so in the manuscript text, or remove these references and replace them with relevant current references. Any changes to the reference list should be mentioned in the rebuttal letter that accompanies your revised manuscript. If you need to cite a retracted article, indicate the article’s retracted status in the References list and also include a citation and full reference for the retraction notice.


[Note: HTML markup is below. Please do not edit.]

# Reviewers' comments:

Reviewer's Responses to Questions

**Comments to the Author**

# 1. Is the manuscript technically sound, and does the data support the conclusions?

The manuscript must describe a technically sound piece of scientific research with data that supports the conclusions. Experiments must have been conducted rigorously, with appropriate controls, replication, and sample sizes. The conclusions must be drawn appropriately based on the data presented.

Reviewer #1: Yes

Reviewer #2: Partly

# 2. Has the statistical analysis been performed appropriately and rigorously?

Reviewer #1: Yes

Reviewer #2: Yes

# 3. Have the authors made all data underlying the findings in their manuscript fully available?

The [PLOS Data policy](http://www.plosone.org/static/policies.action#sharing) requires authors to make all data underlying the findings described in their manuscript fully available without restriction, with rare exception (please refer to the Data Availability Statement in the manuscript PDF file). The data should be provided as part of the manuscript or its supporting information, or deposited to a public repository. For example, in addition to summary statistics, the data points behind means, medians and variance measures should be available. If there are restrictions on publicly sharing data—e.g. participant privacy or use of data from a third party—those must be specified.

Reviewer #1: Yes

Reviewer #2: Yes

# 4. Is the manuscript presented in an intelligible fashion and written in standard English?

PLOS ONE does not copy edit accepted manuscripts, so the language in submitted articles must be clear, correct, and unambiguous. Any typographical or grammatical errors should be corrected at revision, so please note any specific errors here.

Reviewer #1: Yes

Reviewer #2: Yes

# 5. Review Comments to the Author

Please use the space provided to explain your answers to the questions above. You may also include additional comments for the author, including concerns about dual publication, research ethics, or publication ethics. (Please upload your review as an attachment if it exceeds 20,000 characters)

## Reviewer #1:

Dear author,

I found your study topic to be of significance and the findings are of interest to the wider readership. The description of methods and discussion of the results are in adequate detail. I do have the following question and recommendation:

1) Is there a reason why work that does not involve sex has been described as "Straight work"? Is this an objective term? If not, I recommend using a more objective term/phrase. **- This was how respondents described their non-sex work employment in other studies. I have added clarification and mostly removed these.**

2) Paragraph 3 under Conclusions (page 359) makes an inference about sex workers studied in this work not being a captive workforce. Did the present paper study whether the workers were captive or coerced? One of the objective ways to measure this would be interviewing using direct outreach, which was not carried out in this study. It also seems unlikely that the workers who are captive or are coerced, would be advertised online. So if this was not measured, I would advise against making inferences on this subject. **- I have removed this sentence.**

All the best. **- Thank you for taking the time to review this work.**

## Reviewer #2:

I would like to express my appreciation for the effort devoted to the study on sex worker/sex worker time. This issue is extremely important and its approach provides a valuable contribution to the understanding of the labour rights of this population.The study on sex work time of sex workers is a significant contribution to academic literature, addressing a crucial issue that has historically been marginalized. However, a closer review highlights specific areas where methodology and the presentation of results can be strengthened to ensure the robustness and applicability of the conclusions. **- Thank you for taking the time to review this work.**

Regarding the need for greater methodological clarity, it is suggested that the study could benefit from specifying the type of research design used. A qualitative, quantitative or mixed approach can influence the interpretation of the results and provide a deeper context about the nature of the phenomenon studied. Also, explicitly detailing the inclusion and exclusion criteria of the sample would allow readers to evaluate the representativeness of the studied population. Transparency in these areas is essential for the internal validity of the study. **- Admittedly the sampling process is complex and I have made this more explicit by adding a flow diagram of the main steps in the Methods. The inclusion criteria are described as well: only advertisers who used at least one URL as a contact who were active in Aug/Sept 2022 were included. Tracked advertisers were those who represented collectives and provided information on individual workers. Workers counted were all those listed by advertisers on the advertisers’ sites.**

As for the need to consider contextual variables, the research could benefit from a more holistic approach, integrating sociological theories that address the intersectionality of factors such as migration, criminalization, and unemployment in the lives of sex workers. The lack of specific classification of the sites and population studied may limit the generalization of the results. In this sense, a more detailed taxonomy could be extracted from methodologies used in previous studies that have addressed diversity in the sex work industry). In addition, the presentation of the self in everyday life could offer valuable perspectives for understanding social and work dynamics in specific environments. **- Contextual variables are difficult to measure from archival materials therefore I have limited the discussion to what can be measured and avoid too much speculation about the data. Certainly there is a need for follow up work as I believe many of the shorter-term workers represented in the data have not been well studied. Also, I do classify the sites in the Working Collectively subsection of Results based on previous research. Most of these collectives are licensed businesses (83%) based on their website data. I mention another study based on this data that provides a more holistic view of the data in the Materials and Methods section** (Kennedy, 2023). **Interested readers are invited to read that for more information.**

In ethical terms, in the review of the report, questions arise related to the ethical issue, specifically regarding the management and detection of anomalous behaviors, such as trafficking in white women or child exploitation, in the framework of the research. I would like to have more details about the ethical protocols established to deal with these sensitive situations. In particular, I would like to know whether complaints were made to the competent authorities in cases where possible indicators of trafficking in women or child exploitation were detected. Understanding how these ethical issues were handled is essential to ensuring the integrity and social responsibility of the study. **- Keep in mind that this research is done under the guidance of workers who are currently active in the industry. The site used as the original ad source has strict policies against human trafficking and underage advertisers. Hence, none of the sampled advertisers used being under age as a marketing tool. While it is possible that there may have been workers who were coerced - which can be the case in other forms of work - there was no indication of this in the data. Of course, if there were unequivocal indicators of coercion I would have contacted the relevant authorities. Keep in mind there has been little if any work done to empirically validate whether trafficking can be identified using online materials. See for example** (Boecking et al., 2015)**.**

In conclusion, the integration of these suggestions together with a more robust theoretical and methodological basis will strengthen the quality and relevance of the study, allowing a deeper and more nuanced understanding of sex workers' sex work time.

**References**

Boecking, B., Miller, K., Barnes, M., Boecking, B., & Kennedy, E. (2015). Leveraging publicly available data to discern patterns of human-trafficking activity. *Journal of Human Trafficking*, *1*(1), 65–85. https://doi.org/10.1080/23322705.2015.1015342

Kennedy, L. (2023). *Power users: Canadian sex workers’ use of technology post COVID*. SocArXiv. https://doi.org/10.31235/osf.io/u5kd2

# 6. PLOS authors have the option to publish the peer review history of their article ([what does this mean?](https://journals.plos.org/plosone/s/editorial-and-peer-review-process#loc-peer-review-history)). If published, this will include your full peer review and any attached files.

If you choose “no”, your identity will remain anonymous but your review may still be made public.


**Do you want your identity to be public for this peer review?** For information about this choice, including consent withdrawal, please see our [Privacy Policy](https://www.plos.org/privacy-policy).

Reviewer #1: **Yes:** Nitya Kumar

Reviewer #2: No

[NOTE: If reviewer comments were submitted as an attachment file, they will be attached to this email and accessible via the submission site. Please log into your account, locate the manuscript record, and check for the action link "View Attachments". If this link does not appear, there are no attachment files.]

While revising your submission, please upload your figure files to the Preflight Analysis and Conversion Engine (PACE) digital diagnostic tool, <https://pacev2.apexcovantage.com/>. PACE helps ensure that figures meet PLOS requirements. To use PACE, you must first register as a user. Registration is free. Then, login and navigate to the UPLOAD tab, where you will find detailed instructions on how to use the tool. If you encounter any issues or have any questions when using PACE, please email PLOS at figures@plos.org. Please note that Supporting Information files do not need this step.

*In compliance with data protection regulations, you may request that we remove your personal registration details at any time.* [*(Remove my information/details)*](https://www.editorialmanager.com/pone/login.asp?a=r)*. Please contact the publication office if you have any questions.*
